# Case Report: Safety analysis of blinatumomab consolidation therapy in two cases of acute B-cell lymphoblastic leukemia in complete remission complicated with immune effector cell-associated neurotoxicity syndrome

**DOI:** 10.3389/fonc.2026.1743310

**Published:** 2026-02-23

**Authors:** Yixuan Ma, Luyang Chang, Ruihua Mi, Lin Wang, Lin Chen, Xudong Wei

**Affiliations:** Department of Hematology, The Affiliated Cancer Hospital of Zhengzhou University & Henan Cancer Hospital, Zhengzhou, China

**Keywords:** acute B-cell lymphoblastic leukemia, bispecific T-cell engager, blinatumomab, consolidation therapy, immune effector cell-associated neurotoxicity syndrome

## Abstract

**Background:**

Blinatumomab, a bispecific T-cell engager (BiTE), has significantly improved the efficacy of B-ALL treatment. However, its association with immune effector cell-associated neurotoxicity syndrome (ICANS) has garnered increasing attention. This study analyzes two cases of ICANS to explore the challenges in recognizing clinical symptoms and standardizing management.

**Case presentation:**

Case 1 involved a 69-year-old male with B-ALL, harboring *TP53* and *DNMT3A* mutations, who developed grade 2 ICANS following blinatumomab treatment. Therapy was continued without interruption, and symptoms resolved with levetiracetam intervention. Case 2 was a 29-year-old male with B-ALL and *ETV6::RUNX1* fusion gene, who developed grade 4 ICANS (seizures, impaired consciousness, epileptic episodes) on day 4 of blinatumomab therapy. The patient required intensive care and comprehensive intervention, leading to symptom resolution but discontinuation of treatment. Neither case exhibited central nervous system infiltration, and cranial CT scans showed no abnormalities.

**Discussion and conclusion:**

ICANS may be associated with cytokine release, blood-brain barrier disruption, and genetic background, presenting with heterogeneous symptoms. Elderly patients or those with specific genetic mutations may face increased risks, necessitating individualized dose adjustments (e.g., stepwise dosing) and close monitoring of neurological functions and cytokines (e.g., IL-6). Early recognition of subtle symptoms (e.g., tremors) combined with corticosteroids and antiepileptic drugs can improve outcomes. The use of blinatumomab requires careful balancing of efficacy and neurotoxicity, particularly in high-risk patients. This case report provides valuable insights for the clinical recognition and management of ICANS.

## 1 Introduction

In recent years, significant progress has been made in the treatment of adult B-acute lymphoblastic leukemia (B-ALL), owing to a deeper understanding of disease biology, innovations in minimal residual disease (MRD) quantification techniques, refinements in pediatric-inspired chemotherapy regimens, and the introduction of novel immune-targeted agents. Among these, blinatumomab stands out as the most representative immunotherapeutic drug, having demonstrated encouraging efficacy outcomes in patients with relapsed/refractory disease and those with MRD-positive or MRD-negative status. It has been widely adopted in clinical practice globally and is recommended by authoritative guidelines.

Blinatumomab is a novel bispecific T-cell engager (BiTE) that targets CD19 on B-cells and CD3 on T-cells (1, 2). It facilitates direct contact between T-cells and tumor cells, thereby activating the cytotoxic potential of T-cells (3). Furthermore, blinatumomab triggers intrinsic T-cell signaling, leading to the expression of key activation markers such as CD69 and CD25, upregulation of the adhesion molecule CD2, and transient release of inflammatory cytokines. These events collectively result in comprehensive T-cell activation and proliferation, ultimately leading to tumor cell destruction (3).

However, with increasing clinical use in China, reports of immune effector cell-associated adverse reactions have gradually emerged, among which Immune Effector Cell-Associated Neurotoxicity Syndrome (ICANS) has become a significant concern. ICANS is a neurological side effect associated with immunotherapy, with symptoms including headache, confusion, hallucinations, tremors, ataxia, aphasia, behavioral changes, visual or auditory hallucinations, motor dysfunction, seizures, cerebral edema, and even death (4, 5). Its pathogenesis is not fully understood, though previous studies suggest that endothelial activation, induced either by treatment or the disease itself, disrupts the blood-brain barrier. Activated T-cells stimulated by blinatumomab release large quantities of cytokines (e.g., IL-6, IFN-γ), which cross the compromised blood-brain barrier and trigger central nervous system inflammation. This inflammatory cascade alters cortical and subcortical brain function, leading to diffuse cerebral edema (6). The occurrence of ICANS not only affects patients’ quality of life but may also necessitate treatment interruption, thereby impacting overall therapeutic progress and outcomes. Thus, timely recognition and management of this neurotoxicity syndrome are crucial.

This study aims to provide a detailed report of two cases of ICANS, combined with a review of existing literature, to enhance clinical awareness of this adverse reaction and facilitate early identification and effective management.

## 2 Case presentation

### 2.1 Case 1

A 69-year-old male was diagnosed with acute lymphoblastic leukemia (B-cell type). His medical history included postoperative pituitary adenoma and hypothyroidism. He received chemotherapy with rituximab combined with the Hyper-CVAD (A) regimen and achieved complete remission (CR). From November 2022 to December 2023, he underwent multiple cycles of consolidation therapy. In February 2024, disease relapse was confirmed via bone marrow smear, which revealed 78% blasts and immature lymphocytes. Flow cytometry detected 11.32% abnormal immature B lymphocytes expressing CD34, CD10, CD22 (92.67% positivity), CD38, HLA-DR, cCD79a, CD123, and cTDT, with weak expression of CD19 and CD20. Chromosome analysis showed an abnormal karyotype with poor morphology: 47, XY, +Y[3]/36, X, -Y, -2, -7, -7, -12, -13, -15, -16, -17, -20[cp2]/46, XY[5], and fluorescence *in situ* hybridization (FISH) was negative for the *BCR::ABL1* fusion gene (Philadelphia chromosome-negative). RNA sequencing identified mutations in *TP53* c.G818A, *WT1* c.C1387T, and *DNMT3A* c.C1903T.

In March 2024, he received one cycle of inotuzumab ozogamicin and achieved remission again. In June 2024, he received another cycle of inotuzumab ozogamicin as consolidation therapy. During this period, lumbar puncture and intrathecal chemotherapy were performed, with no abnormal cells detected in the cerebrospinal fluid. From July to October 2024, he received three cycles of blinatumomab treatment (first cycle: dose gradually increased to 28 μg/day for 27 days; second cycle: 28 μg/day for 15 days; third cycle: 28 μg/day for 11 days). On the first day of the third cycle, the patient developed transient hand and foot tremors and mild drowsiness but could be awakened softly. Cranial CT showed no structural abnormalities. Based on ASTCT consensus criteria, he was diagnosed with grade 2 ICANS. Symptoms resolved after treatment with oral levetiracetam 500 mg twice daily. Blinatumomab was not discontinued, and the patient did not develop fever, generalized seizures, or severe impaired consciousness. Currently, the patient remains in complete remission with minimal residual disease (MRD)-negative status and continues to receive blinatumomab as maintenance therapy.

### 2.2 Case 2

A 29-year-old male was diagnosed with acute lymphoblastic leukemia (B-cell type) with *ETV6::RUNX1* fusion gene, the patient did not harbor the *BCR::ABL1* fusion gene. In August 2023, he began chemotherapy with the VDCLP regimen and achieved complete remission after one cycle. From September to November 2023, he received three cycles of consolidation chemotherapy, followed by one cycle of blinatumomab treatment in November 2023. During this period, the patient experienced mild upper limb tremors, which resolved spontaneously. From January to May 2024, he received multiple cycles of consolidation chemotherapy. Repeated lumbar punctures and intrathecal chemotherapy were performed, with no abnormal cells detected in the cerebrospinal fluid. In July 2024, he was admitted to our hospital for evaluation. The primary disease remained in complete remission with minimal residual disease (MRD)-negative status. Blinatumomab treatment was initiated at 28 μg/day without dose escalation. On the fourth day of treatment, he suddenly developed convulsions in the limbs and head, upward deviation of the eyes, epileptic seizures, and loss of consciousness, accompanied by hypernatremia (149 mmol/L) and elevated myoglobin (221.4 ng/mL). Cranial CT showed no structural abnormalities. Based on ASTCT consensus criteria, he was diagnosed with grade 4 ICANS. Blinatumomab was immediately discontinued, and the patient received intravenous dexamethasone (10 mg every 6 hours for 5 days, followed by a rapid taper) for anti-inflammatory purposes, levetiracetam (500 mg twice daily) for seizure control, and diazepam for sedation. Tocilizumab was not administered because the patient did not exhibit symptoms of concurrent severe CRS, and guidelines suggest limited benefit of tocilizumab for isolated ICANS (7). After symptom control, the patient developed agitation, confusion, cognitive decline, and dysuria, necessitating transfer to the intensive care unit. His ICANS symptoms resolved after treatment with sedation, antiepileptic drugs, anti-inflammatory agents, and electrolyte adjustment. On the fifth day of treatment (within 24 hours of ICANS onset), laboratory tests revealed serum IL-6 at 5.77 pg/mL, IFN-γ at 4.17 pg/mL, and ferritin at 681.00 ng/mL. These relatively modest elevations, despite the clinical severity of ICANS, suggest that the neurotoxicity may involve localized blood-brain barrier disruption or focal neuroinflammation rather than a systemic cytokine storm Lumbar puncture and intrathecal chemotherapy were performed, with no abnormal cells detected in the cerebrospinal fluid. Due to the severe adverse reaction, blinatumomab was not resumed.

## 3 Discussion

As the only commercially available bispecific T-cell engager in the field of B-cell acute lymphoblastic leukemia (B-ALL), blinatumomab has been demonstrated in multiple clinical studies to provide significant survival benefits and manageable safety for patients with B-ALL, profoundly altering the treatment landscape for relapsed/refractory (R/R) and minimal residual disease (MRD)-positive B-ALL (8). With the expanding clinical research, approved indications, and increased clinical application of blinatumomab, the issue of immune effector cell-associated neurotoxicity syndrome (ICANS) induced by this agent has become increasingly prominent. Previously considered part of cytokine release syndrome (CRS), neurotoxicity is now recognized as a distinct entity due to its unique temporal pattern and response to interventions (9). Lee et al. proposed defining ICANS as a disorder involving the central nervous system following any immunotherapy, resulting from the activation and involvement of endogenous or infused T cells and/or other immune effector cells (10). Symptoms and signs are progressive and may include aphasia, altered consciousness, cognitive impairment, motor weakness, seizures, and cerebral edema. ICANS applies to any therapy involving immune effector cells, not limited to CAR-T cells. Thus, with the increasing clinical use of blinatumomab, ICANS, due to its complex and insidious symptoms and frequent co-occurrence with CRS, has become an easily overlooked yet critical safety concern in clinical practice.

In real-world studies, the incidence of grade ≥3 ICANS is 7%, with a median time of onset at day 9 after the initiation of infusion, most neurological events resolve spontaneously (8). ICANS may occur concurrently with CRS or shortly after its resolution. It is typically self-limiting, with symptoms lasting 5 to 17 days (4, 5). For grade 2 or higher ICANS, dexamethasone 10 mg every 6 to 12 hours is recommended, followed by a taper over 2 to 5 days. In patients at high risk of seizures, levetiracetam 500 mg twice daily is advised for prophylaxis. If clinical seizures occur, antiepileptic drugs should be adjusted to therapeutic doses (3). In contrast, CD19-targeted CAR-T therapy has been associated with a 26%–36% incidence of grade ≥3 ICANS in a meta-analysis, and ICANS is one of the leading causes of CAR-T-related mortality, accounting for 15.1% of cases (11). Therefore, accurate assessment and timely management of ICANS in clinical practice can mitigate adverse events associated with immunotherapy, thereby maximizing its clinical benefits while ensuring safety.

The two cases of ICANS we reported occurred during complete remission (CR) of the primary disease. Case 1 involved an elderly patient receiving blinatumomab who developed grade 2 ICANS despite a low tumor burden (MRD 0.18%). While advanced age is often clinically perceived as a potential risk factor due to decreased neurological reserve, the specific impact of age on blinatumomab-related ICANS remains controversial (6). Indeed, the TOWER study reported a lower incidence of severe ICANS in elderly patients compared to younger cohorts, while another study showed no significant difference in severe ICANS incidence between those aged 65 and older versus other age groups (2, 12). This discrepancy might be explained by more proactive preventive measures or vigilant dose-escalation strategies typically employed in older populations. Our observation in Case 1 underscores that while age itself may not statistically increase the incidence of high-grade ICANS, individualized monitoring remains paramount for elderly patients to prevent symptom escalation. Notably, Case 1 experienced a ICANS episode on day 42, representing a substantially delayed and atypical temporal pattern compared to the standard onset reported in TOWER trials (2). This delayed presentation may be attributed to cumulative drug exposure across multiple cycles or heightened individual immune response variability. Furthermore, concurrent subtle factors, such as minor metabolic fluctuations or subclinical infections, might have lowered the neurological threshold. This case highlights the importance of sustained, long-term neurological surveillance even beyond the initial high-risk period of the first cycle. Additionally, the concurrent presence of *TP53* and *DNMT3A* mutations raises a hypothetical possibility of a genetic predisposition to neurotoxicity. However, it must be emphasized that direct clinical evidence linking these specific mutations to ICANS risk is currently lacking, and this remains an area for future systematic investigation. Tremors, as an atypical manifestation of ICANS, can easily be overlooked and require early identification through electroencephalography and cytokine monitoring. The TOWER study showed a lower incidence of ICANS in elderly patients (9.4% vs. 1.8%), but with milder symptoms, indicating possible differences in dose tolerance (2). Case 1 exhibited low CD19 expression at relapse. Although blinatumomab may still hold therapeutic value for patients with partial or low CD19 expression, reduced antigen density could diminish efficacy and increase the risk of clonal evolution or antigen loss (13, 14). Therefore, during ongoing maintenance therapy, clinicians must strictly and frequently monitor CD19 expression levels and MRD to detect signs of immune escape early and adjust treatment strategies in a timely manner.

In Case 2, a young patient presented mild upper limb tremors during the first cycle of blinatumomab treatment but progressed typical ICANS symptoms (seizures, impaired consciousness, etc.) during the second exposure. This progression suggests that the severity of neurotoxicity may correlate with the intensity of the individual immune response. Furthermore, it is essential to consider differential diagnoses for the neurological events in Case 2. While hypernatremia (149 mmol/L) and elevated myoglobin could potentially exacerbate seizure activity or reflect metabolic encephalopathy (15, 16), the diagnosis of ICANS was based on the close temporal correlation with blinatumomab infusion and the resolution of symptoms following standard ICANS management. The myoglobin elevation was likely secondary to the generalized convulsions (15). This suggests that when ICANS occurs outside the typical timeframe, a more comprehensive etiological evaluation is necessary to identify and address all potential aggravating factors (17). It could also be speculated whether the *ETV6::RUNX1* fusion gene influences the immune microenvironment in the central nervous system, potentially contributing to the severity of ICANS. Currently, there is no established evidence to support enhanced CNS penetration of blinatumomab in the presence of this fusion gene, and the causal relationship remains speculative.

These two cases highlight the heterogeneity of ICANS, where initial mild neurological adverse reactions may be overlooked but can later manifest as more severe ICANS. Early recognition of these neurological symptoms and timely adjustment of treatment strategies are essential. Corticosteroids, tocilizumab, and antiepileptic drugs are effective interventions for managing ICANS. Moreover, in case 1, symptoms resolved after treatment with oral levetiracetam 500 mg twice daily. Although this management deviates from the ASTCT guidelines, which recommend dexamethasone for grade 2 or higher ICANS, the patient’s symptoms were mild and rapidly controlled (6, 7). This suggests that while individualized management may be sufficient in select mild cases, strict adherence to corticosteroid protocols is generally prioritized to prevent progression. This case highlights the importance of balancing clinical judgment with standardized guidelines under close neurological surveillance.

In summary, blinatumomab demonstrates remarkable efficacy in the treatment of B-ALL, but ICANS remains a significant complication, presenting as a spectrum of neurotoxicities ranging from mild tremors to seizures and even death. The underlying mechanisms involve cytokine storms and blood-brain barrier disruption, necessitating management tailored to individual patient characteristics. Patients with high tumor burden or specific genetic mutations should receive full-dose blinatumomab with caution, and dose escalation should be prioritized. Close monitoring of cytokines (e.g., IL-6) and neurological function during treatment is essential for early intervention. Through these measures, the efficacy and safety of blinatumomab can be optimally balanced, providing patients with improved treatment options. Through in-depth analysis of these two cases, we aim to elucidate the clinical features and treatment correlations of ICANS, offer insights for clinicians, and discuss considerations for future blinatumomab therapy.

## Data Availability

The original contributions presented in the study are included in the article/supplementary material. Further inquiries can be directed to the corresponding author.

## References

[1] Loffler A Gruen M Wuchter C Schriever F Kufer P Dreier T . Efficient elimination of chronic lymphocytic leukaemia B cells by autologous T cells with a bispecific anti-CD19/anti-CD3 single-chain antibody construct. Leukemia. (2003) 17:900–9. doi: 10.1038/sj.leu.2402890, PMID: 12750704

[2] Kantarjian H Stein A Gokbuget N Fielding AK Schuh AC Ribera JM . Blinatumomab versus chemotherapy for advanced acute lymphoblastic leukemia. N Engl J Med. (2017) 376:836–47. doi: 10.1056/NEJMoa1609783, PMID: 28249141 PMC5881572

[3] Prigozy TI Sieling PA Clemens D Stewart PL Behar SM Porcelli SA . The mannose receptor delivers lipoglycan antigens to endosomes for presentation to T cells by CD1b molecules. Immunity. (1997) 6:187–97. doi: 10.1016/s1074-7613(00)80425-2, PMID: 9047240

[4] Santomasso BD Nastoupil LJ Adkins S Lacchetti C Schneider BJ Anadkat M . Management of immune-related adverse events in patients treated with chimeric antigen receptor T-cell therapy: ASCO guideline. J Clin Oncol. (2021) 39:3978–92. doi: 10.1200/JCO.21.01992, PMID: 34724386

[5] Jain MD Smith M Shah NN . How I treat refractory CRS and ICANS after CAR T-cell therapy. Blood. (2023) 141:2430–42. doi: 10.1182/blood.2022017414, PMID: 36989488 PMC10329191

[6] Danish H Santomasso BD . Neurotoxicity biology and management. Cancer J. (2021) 27:126–33. doi: 10.1097/PPO.0000000000000507, PMID: 33750072

[7] Lee DW Santomasso BD Locke FL Ghobadi A Turtle CJ Brudno JN . ASTCT consensus grading for cytokine release syndrome and neurologic toxicity associated with immune effector cells. Biol Blood Marrow Transplant. (2019) 25:625–38. doi: 10.1016/j.bbmt.2018.12.758, PMID: 30592986 PMC12180426

[8] Union for China Leukemia of Chinese Society of Clinical, O Union for Lymphoma Investigators of Chinese Society of Clinical, O Chinese Society of Hematology, C. M. A Chinese Hematology Association, C. M. D. A . Chinese consensus for the bispecific T cell engager in the treatment of acute lymphoblastic leukemia. Zhonghua Xue Ye Xue Za Zhi. (2024) 45:629–36. doi: 10.3760/cma.j.cn121090-20240528-00194, PMID: 39231766 PMC11388125

[9] Fu CC Wang RJ Wu DP . Interpretation of ASTCT consensus responses by chimeric antigen receptor T cell therapy CRS/ICANS--review. Zhongguo Shi Yan Xue Ye Xue Za Zhi. (2021) 29:1982–6. doi: 10.19746/j.cnki.issn.1009-2137.2021.06.051, PMID: 34893146

[10] Lee DW Gardner R Porter DL Louis CU Ahmed N Jensen M . Current concepts in the diagnosis and management of cytokine release syndrome. Blood. (2014) 124:188–95. doi: 10.1182/blood-2014-05-552729 PMC4093680 24876563

[11] Lei W Xie M Jiang Q Xu N Li P Liang A . Treatment-related adverse events of chimeric antigen receptor T-cell (CAR T) in clinical trials: A systematic review and meta-analysis. Cancers (Basel). (2021) 13:3912. doi: 10.3390/cancers13153912, PMID: 34359816 PMC8345443

[12] Topp MS Gokbuget N Stein AS Zugmaier G O’Brien S Bargou RC . Safety and activity of blinatumomab for adult patients with relapsed or refractory B-precursor acute lymphoblastic leukaemia: a multicentre, single-arm, phase 2 study. Lancet Oncol. (2015) 16:57–66. doi: 10.1016/S1470-2045(14)71170-2, PMID: 25524800

[13] Qi Y Liu H Li X Shi Y Mu J Li J . Blinatumomab as salvage therapy in patients with relapsed/refractory B-ALL who have failed/progressed after anti-CD19-CAR T therapy. Ann Med. (2023) 55:2230888. doi: 10.1080/07853890.2023.2230888, PMID: 37417690 PMC10332179

[14] Myers RM Taraseviciute A Steinberg SM Lamble AJ Sheppard J Yates B . Blinatumomab nonresponse and high-disease burden are associated with inferior outcomes after CD19-CAR for B-ALL. J Clin Oncol. (2022) 40:932–44. doi: 10.1200/JCO.21.01405, PMID: 34767461 PMC8937010

[15] Pokrzywnicki W Grzeszczak W Motta E Rosciszewska D Kapustecki J Kochanska A . Level of myoglobin in serum of patients with epilepsy after generalized tonic-clonic seizure. Wiad Lek. (1994) 47:267–73., PMID: 7941577

[16] Adrogue HJ Madias NE . Hypernatremia. N Engl J Med. (2000) 342:1493–9. doi: 10.1056/NEJM200005183422006, PMID: 10816188

[17] Neelapu SS Tummala S Kebriaei P Wierda W Gutierrez C Locke FL . Chimeric antigen receptor T-cell therapy - assessment and management of toxicities. Nat Rev Clin Oncol. (2018) 15:47–62. doi: 10.1038/nrclinonc.2017.148, PMID: 28925994 PMC6733403

